# Use of Molecular Genetic Methods to Reduce the Risk of Incorrect Identification of Fish Strains in Brazilian Aquaculture

**DOI:** 10.3389/fgene.2021.720736

**Published:** 2021-12-09

**Authors:** Luana Maria Deoclécio da Silva, Fernanda Dotti do Prado, Diogo Teruo Hashimoto, José Augusto Senhorini, Fausto Foresti, Fabio Porto-Foresti

**Affiliations:** ^1^ Laboratório de Genética de Peixes, Faculdade de Ciências, Departmento de Ciências Biológicas, Universidade Estadual Paulista, Bauru, Brazil; ^2^ Laboratório de Genética em Aquicultura e Conservação, Centro de Aquicultura, Universidade Estadual Paulista, Jaboticabal, Brazil; ^3^ Centro Nacional de Pesquisa e Conservação da Biota Aquática Continental (CEPTA-ICMBIO), Pirassununga, Brazil; ^4^ Laboratório de Biologia e Genética de Peixes, Instituto de Biociências, Universidade Estadual Paulista, Botucatu, Brazil

**Keywords:** aquaculture, catFISH, interspecific hybridization, molecular markers, PCR multiplex

## Abstract

The identification of fish species using traditional methods is generally based only on morphological characteristics and these methods are currently under review. This kind of identification of hybrid fishes solely based on their morphologies may be unreliable, especially when the samples include juveniles and post-F1 lineage fishes. Therefore, in the present study, we used molecular markers to accurately identify the fish species of economic interest that are used as materials in the projects developed in research institutions. We evaluated six lots of fishes sampled from different research centers, containing a total of 84 specimens acquired from private fish farms that were considered to be the representatives of pure species. Genetic analyses of all the specimens revealed that, globally, 22 samples (26.2%) were interspecific hybrids, while 20 (90.9%) samples were surprisingly characterized as post-F1 hybrids. This result confirms that hybrids are sold in markets without adequate labeling and also indicates the lack of proper control of the commercialization and management of stocks and products in fish farms. In addition, we determined that molecular diagnosis was an extremely effective and necessary method to test the reliability of biological materials currently used in scientific research.

## Introduction

There is an extensive diversity of fish species in the Neotropical region, including Brazil (Vari and Malabarba, 1998; Reis et al., 2016). Despite the presence of a large number of species of economic interest and great potential for their cultivation, consistent data and information on the biology, reproduction, genetics, nutrition, and zootechnical management of these species are still lacking. Many laboratories and research centers are currently working to increase their production chain (see Barroso et al., 2013; Mastrochirico-Filho et al., 2016). These laboratories usually acquire their specimens from private fish farms, which maintain their own stocks of matrices and store their production for sale. However, with the increasing production of interspecific hybrids, the purity of individuals used as study models has become uncertain.

In recent years, the increase in world demand for fish products has led to a significant expansion of the fish farming sector. This advance is associated with the development of several genetic improvement techniques applied to production, including the artificial interspecific hybridization (Bartley et al., 2001; Porto-Foresti et al., 2011; Porto-Foresti et al., 2013; Hashimoto et al., 2016). In Brazil, the production of native fish and their hybrids has been a great target of agricultural investment, mainly for the catfish species *Pseudoplatystoma reticulatum* (cachara), *Pseudoplatystoma corruscans* (pintado) and *Leiarius marmoratus* (jundiá) (Campos, 2010; Hashimoto et al., 2012a). The national aquaculture sector has increasingly encouraged the large-scale cultivation of these hybrids, as they have more advantageous commercial characteristics when compared to parental species, such as higher growth rates and better meat quality (Crepaldi et al., 2006; Campos, 2010). But, there are some risks associated with the mismanagement of artificial hybrids and their effects on biodiversity conservation and sustainable food production.

The morphological similarity between hybrids and pure species in cultivated stocks can result in mixtures and occasional introgressions, sometimes resulting in serious problems in the maintenance of populations and species (Allendorf and Leary, 1988; Epifanio and Philipp, 2001; Rosenfield et al., 2004). Accidental and uncontrolled hybridization can generate undesirable results in hybrid progenies, such as loss of product quality and reduced growth performance, as well as compromising the genetic integrity of the species (Rahman et al., 2013). According to (Toledo-Filho et al., 1994), the problems could be minimized and even avoided with the knowledge of the genetic profile of the animals involved in the crossings. In this case, effective programs for the characterization and genetic monitoring of stocks must be essential in fish farms, ensuring the correct labeling of parental lines and commercialized products (Toledo-Filho et al., 1994, 1998; Porto-Foresti et al., 2008; Hashimoto et al., 2012a).

Differentes DNA markers have been developed and successfully applied for the identification of fish hybrids species (Hashimoto et al., 2011; Prado et al., 2011; Hashimoto et al., 2012a; Hashimoto et al., 2016; Yabu et al., 2018). Here, we used some of these markers to evaluate the correct identity of catfish species that have been used as material in projects developed in research institutions. Our aim was to verify the purity of the species used in these projects and assess the potential of previous molecular diagnosis to prevent the possible misuse of hybrids as biological material in research.

## Methods

All analyses were conducted at the Fish Genetics Laboratory of the Department of Biological Sciences of the Faculty of Sciences of Bauru or Universidade Estadual Paulista (UNESP), which has expertise in the development of specific genetic markers for Neotropical fish species. We obtained a total of 84 biological samples of *P. corruscans* (pintado), *P. reticulatum (cachara)* and *L. marmoratus (jundiá)* from five different research centers, hereinafter referred to as institution A, B, C, D and E (Table 1). All samples evaluated in this study were acquired from private Brazilian fish farms, commercially classified by external morphology as pure species. The samples were diagnosed using the multiplex polymerase chain reaction (Multiplex PCR) following the conditions described by Prado et al. (2011) and Hashimoto et al. (2016) for the identification of species belonging to the order Siluriformes. Here we used one mitochondrial marker (16S ribosomal RNA) and three single nuclear genes: RAG2 (nuclear recombination activating gene 2), EF1α (elongation factor 1-alpha) and glob (β-globin). The amplified products were submitted to electrophoresis in a 1.5% agarose gel and visualized in an ultraviolet (UV) transluminator. According to the genotyping performed through the combine results of nuclear markers, the samples were classified into one of three categories: 1) pure species, if all markers had homozygous genotypes for the initially classified species; 2) F1 hybrid, if all markers had heterozygous genotypes; and 3) post-F1 hybrid presenting combined homozygous and heterozygous genotypes for the nuclear markers. The mitochondrial gene was used to identify the maternal species involved in crosses.

**TABLE 1:** Institution of origin, number of individuals and morphological (phenotypic) identification of all samples analyzed in the current study.

Institution	Number of individuals	Morphological identification
A	49	*Pseudoplatystoma reticulatum (cachara)*
B	5	*Pseudoplatystoma reticulatum (cachara)*
C	6	*Pseudoplatystoma corruscans (pintado)*
D	15	*Pseudoplatystoma corruscans (pintado)*
E	9	*Leiarius marmoratus (jundiá)*

## Results and Discussion

**FIGURE 1:**
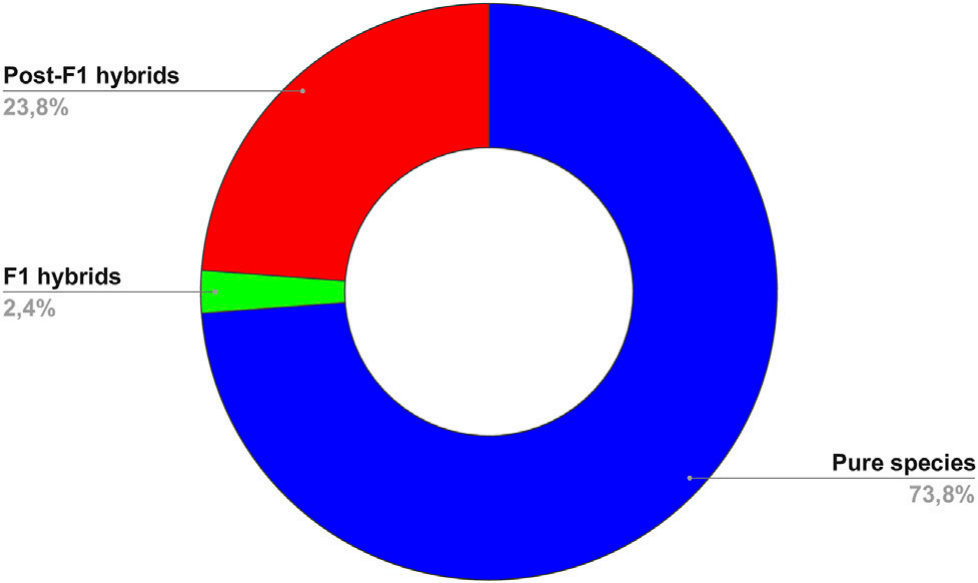
Total percentage of hybrids (F1 and post-F1 generations) among all 84 samples marketed as pure species in private fish farms.

**FIGURE 2:**
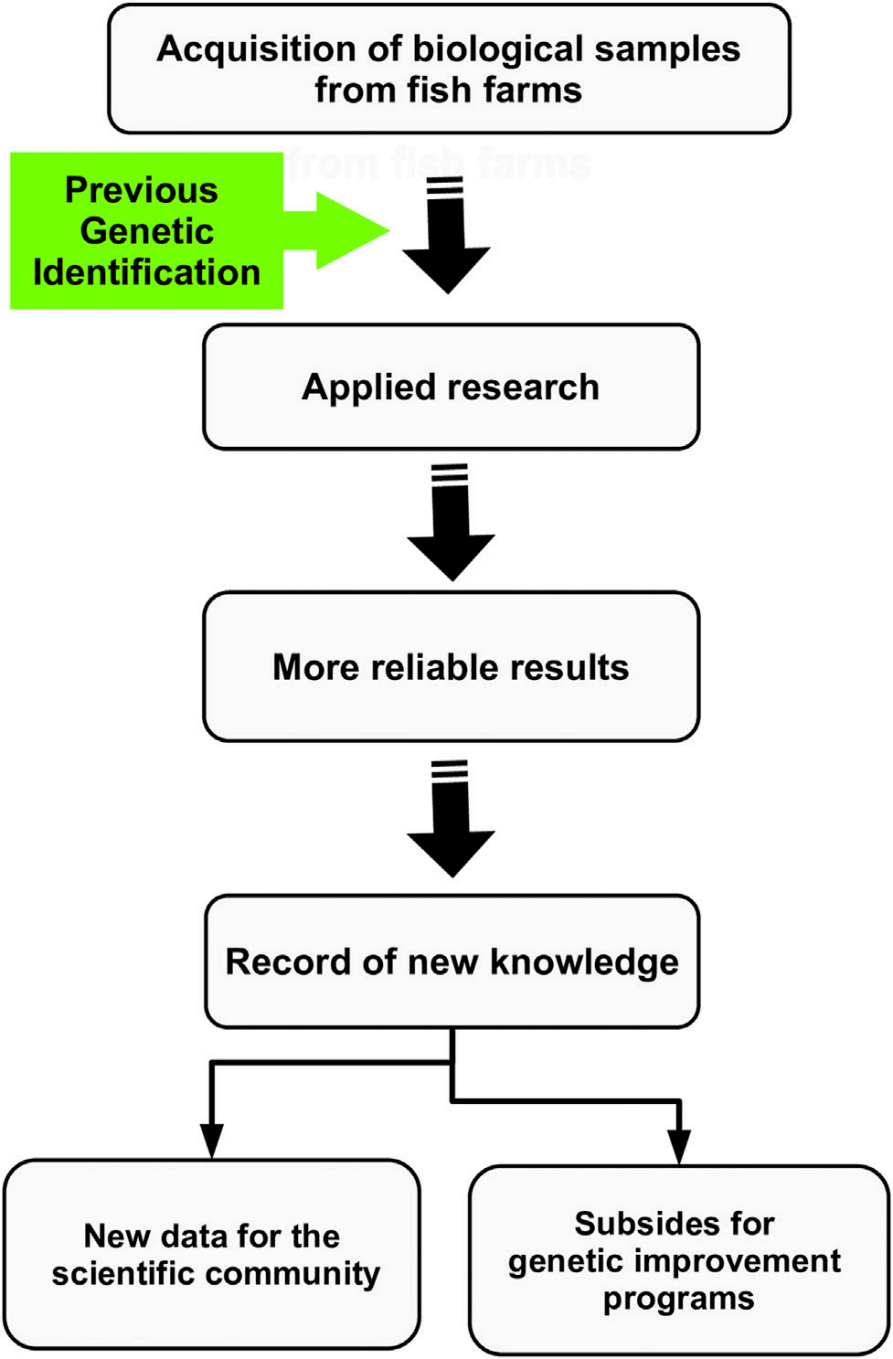
Illustrative scheme demonstrating the importance of previous genetic diagnosis of samples for the correct identification of species and subsidies for research projects and future breeding programs.

Our results showed that of the 84 analyzed samples, 73.80% presented the genetic profile of pure species (confirming their initial classification) and 26.20% were found as hybrids (mislabeled specimens), with most of them belonging to the post-F1 class (23.80%) and 2.40% of individuals probably belonging to a F1 hybrid category (Figure 1). All information used in the molecular diagnostic is available in Supplementary Appendix SAI (Supplementary Data). The samples belonging to institutions A and E were the only ones where we found 100% purity, where the initial characterization of the species was done correctly. However, the initial identification only by morphology was not accurate for the other samples, where we identified 40%, 100% and 83.33% of species as hybrids, referring to institutions B, C and D, respectively (Table 2). More alarming, among all identified hybrids, 90.90% were classified as post-F1 generation, indicating the occurrence of backcrosses in the stocks of origin. Misuse of interspecific hybrid fish as pure species in scientific projects can generate false-positive results and lead to the dissemination of unreliable information about the species. Although hybrids may appear externally similar to pure species, they have divergent biological characteristics (Toledo-Filho et al., 1994). Furthermore, the zootechnical advantages associated with the “hybrid vigor” may be absent in post-F1 generations, due to the loss of heterosis observed in F1 by introgressive hybridization (Hashimoto et al., 2012b). These factors highlight the need for previous molecular diagnosis in studies involving mainly commercial Neotropical fish species. In addition to obtaining more accurate results, previous genetic identification allows access to new knowledge that can serve as subsides for future genetic improvement programs and commercial production of these species (Figure 2).

**TABLE 2:** Genetic diagnosis of catfish species from different research center acquired from private fish farms. N: number of individuals sampled; F1 hybrid: first generation interspecific hybrid; Post-F1 hybrid: advanced interspecific hybrid.

Samples	Morphological identification (N)	Genotype (N)	Hybrids in the samples (%)
A	*Pseudoplatystoma reticulatum* (49)	*P. reticulatum* (49)	0%
		F1 hybrid (0)	
		Post-F1 hybrid (0)	
B	*Pseudoplatystoma reticulatum* (5)	*P. reticulatum* (3)	40%
		F1 hybrid (1)	
		Post-F1 hybrid (1)	
C	*Pseudoplatystoma corruscans* (6)	*P. corruscans* (0)	100%
		F1 hybrid (0)	
		Post-F1 hybrid (6)	
D	*Pseudoplatystoma corruscans* (15)	*P. corruscans* (1)	93.33%
		F1 hybrid (1)	
		Post-F1 hybrid (13)	
E	*Leiarius marmoratus* (9)	*L. marmoratus* (9)	0%
		F1 hybrid (0)	
		Post-F1 hybrid (0)	

The present study demonstrated the effectiveness of molecular markers to assess the reliability of fish farms stocks and products, highlighting the need of correct marking (i.e., genetic identification) for use by the scientific community. Our data confirm that the identification only by external morphology can be imprecise in most cases (institutions B, C and D), reinforcing the importance of previous molecular diagnosis in the characterization of these species. The use of single genes by the Multiplex PCR technique proved to be satisfactory for making this diagnosis, as in addition to producing fast and accurate results, it is a simple and low cost tool. Finally, our results confirm the lack of control in the management and commercialization of fish farm products, with the consequent genetic contamination of breeding stocks and the sale of hybrids without proper labeling.

## Data Availability

The raw data supporting the conclusion of this article will be made available by the authors, without undue reservation.
