# Supplementary material for: Use of Molecular Genetic Methods to Reduce the Risk of Incorrect Identification of Fish Strains in Brazilian Aquaculture
Source: Front Genet. 2021 Dec 9;12:720736. doi: 10.3389/fgene.2021.720736 (PMC8695485; doi:10.3389/fgene.2021.720736)
Supplement: Supplementary file 1 [file DataSheet1.PDF]

Appendix I. Supplementary data.

Phenotypes, genotypes by molecular markers and popular names of samples. Pc: *P. corruscans* marker, Pr: *P. reticulatum* marker, Lm: *Leiarius marmoratus* marker, Cachapinta: F1 hybrid between *P. reticulatum* female and *P. corruscans* male.

**Institution**	**Samples**	**Phenotypes**	**Molecular Markers**												**Genotypes**	**Popular Names**
			**RAG2**			**16S**			**GLOBINA**			**EF1**				
			**Pc**	**Pr**	**Lm**	**Pc**	**Pr**	**Lm**	**Pc**	**Pr**	**Lm**	**Pc**	**Pr**	**Lm**		
A	1-49	*P. reticulatum*	-	x	-	-	x	-	-	x	-	-	x	-	*P. reticulatum*	Cachara
B	1	*P. reticulatum*	-	x	-	-	x	-	-	-	-	-	x	-	*P. reticulatum*	Cachara
	2	*P. reticulatum*	-	x	-	-	x	-	-	x	-	-	-	-	*P. reticulatum*	Cachara
	3	*P. reticulatum*	-	x	-	-	x	-	-	x	-	-	x	-	*P. reticulatum*	Cachara
	4	*P. reticulatum*	x	x	-	-	-	-	x	x	-	x	x	-	F1 HYBRID	-
	5	*P. reticulatum*	-	x	-	-	x	-	x	x	-	-	x	-	Post F1 Hybrid	Cachapinta
C	1	*P. corruscans*	x	-	-	-	x	-	x	-	-	x	-	-	Post F1 Hybrid	Cachapinta
	2	*P. corruscans*	x	-	-	-	x	-	x	-	-	x	-	-	Post F1 Hybrid	Cachapinta
	3	*P. corruscans*	x	x	-	-	x	-	x	-	-	x	-	-	Post F1 Hybrid	Cachapinta
	4	*P. corruscans*	x	-	-	-	x	-	x	-	-	x	-	-	Post F1 Hybrid	Cachapinta
	5	*P. corruscans*	x	-	-	-	x	-	x	-	-	x	-	-	Post F1 Hybrid	Cachapinta
	6	*P. corruscans*	x	-	-	-	x	-	x	-	-	x	-	-	Post F1 Hybrid	Cachapinta
D	1	*P. corruscans*	x	x	-	-	x	-	x	-	-	x	-	-	Post F1 Hybrid	Cachapinta
	2	*P. corruscans*	x	-	-	-	x	-	x	-	-	x	-	-	Post F1 Hybrid	Cachapinta
	3	*P. corruscans*	x	-	-	-	x	-	x	-	-	x	x	-	Post F1 Hybrid	Cachapinta
	4	*P. corruscans*	x	-	-	-	x	-	x	-	-	x	-	-	Post F1 Hybrid	Cachapinta
	5	*P. corruscans*	x	-	-	x	-	-	x	-	-	x	-	-	*P. corruscans*	Pintado
	6	*P. corruscans*	x	x	-	-	x	-	x	-	-	x	-	-	Post F1 Hybrid	Cachapinta
	7	*P. corruscans*	x	x	-	-	x	-	x	x	-	x	-	-	Post F1 Hybrid	Cachapinta
	8	*P. corruscans*	x	x	-	-	x	-	x	x	-	x	x	-	F1 HYBRID	Cachapinta
	9	*P. corruscans*	x	-	-	-	x	-	x	x	-	x	-	-	Post F1 Hybrid	Cachapinta

	10	*P. corruscans*	x	-	-	-	x	-	x	-	-	x	-	-	Post F1 Hybrid	Cachapinta
	11	*P. corruscans*	x	-	-	-	x	-	x	-	-	x	-	-	Post F1 Hybrid	Cachapinta
	12	*P. corruscans*	x	x	-	-	x	-	x	-	-	x	-	-	Post F1 Hybrid	Cachapinta
	13	*P. corruscans*	x	-	-	-	x	-	x	-	-	x	-	-	Post F1 Hybrid	Cachapinta
	14	*P. corruscans*	x	x	-	-	x	-	x	-	-	x	-	-	Post F1 Hybrid	Cachapinta
	15	*P. corruscans*	x	x	-	-	x	-	x	-	-	x	x	-	Post F1 Hybrid	Cachapinta
	1	*L. marmoratus*	-	-	x	-	-	x	-	-	-	-	-	x	*L. mamoratus*	Jundiá
	2	*L. marmoratus*	-	-	x	-	-	x	-	-	-	-	-	x	*L. mamoratus*	Jundiá
	3	*L. marmoratus*	-	-	x	-	-	x	-	-	-	-	-	x	*L. mamoratus*	Jundiá
	4	*L. marmoratus*	-	-	x	-	-	x	-	-	-	-	-	x	*L. mamoratus*	Jundiá
E	5	*L. marmoratus*	-	-	x	-	-	x	-	-	-	-	-	x	*L. mamoratus*	Jundiá
	6	*L. marmoratus*	-	-	x	-	-	x	-	-	-	-	-	x	*L. mamoratus*	Jundiá
	7	*L. marmoratus*	-	-	x	-	-	x	-	-	-	-	-	x	*L. mamoratus*	Jundiá
	8	*L. marmoratus*	-	-	x	-	-	x	-	-	-	-	-	x	*L. mamoratus*	Jundiá
	9	*L. marmoratus*	-	-	x	-	-	x	-	-	-	-	-	x	*L. mamoratus*	Jundiá
